# Evaluation of optimal treatment planning for radiotherapy of synchronous bilateral breast cancer including regional lymph node irradiation

**DOI:** 10.1186/s13014-019-1257-5

**Published:** 2019-04-01

**Authors:** Yeona Cho, Yoon Jin Cho, Won Suk Chang, Jun Won Kim, Won Hoon Choi, Ik Jae Lee

**Affiliations:** Department of Radiation Oncology, Gangnam Severance Hospital, Yonsei University College of Medicine, 211 Eonju-ro, Gangnam-gu, Seoul, 06273 Korea

**Keywords:** Radiotherapy dosage, Breast Cancer, Volumetric-modulated arc therapy, Organs at risk

## Abstract

**Background:**

We evaluated the optimal radiotherapy (RT) plan for synchronous bilateral breast cancer (SBBC), especially treatment plans including the regional lymph node (LN) area.

**Methods:**

This study was conducted using 15 patients with SBBC (5 with small breasts, 5 with large breasts, and 5 who underwent a left total mastectomy). The clinical target volume (CTV) was defined as the volume enveloping the bilateral whole breasts/chest wall and left regional LN area. We established the following plans: 1) volumetric-modulated arc therapy (VMAT)-the only plan using two pairs of partial arcs for the whole target volume, 2) VMAT using one partial arc for the left CTV followed by a 3D tangential technique for the right breast (primary hybrid plan), and 3) VMAT for the left CTV followed by a tangential technique using an automatically calculated prescription dose for the right breast, considering the background dose from the left breast VMAT plan (modified hybrid plan). The Tukey test and one-way analysis of variance were used to compare the target coverage and doses to organs at risk (OARs) of the three techniques.

**Results:**

For target coverage, the VMAT-only and modified hybrid plans showed comparable target coverage in terms of D_mean_ (50.33 Gy vs. 50.53 Gy, *p* = 0.106). The primary hybrid plan showed the largest distribution of the high-dose volume, with V_105%_ of 25.69% and V_110%_ of 6.37% for the planning target volume (PTV) (*p* < 0.001). For OARs including the lungs, heart, and left anterior descending artery, the percentages of volume at various doses (V_5Gy_, V_10Gy_, V_20Gy_, V_30Gy_) and D_mean_ were significantly lower in both the primary and modified hybrid plans compared to those of the VMAT-only plan. These results were consistent in subgroup analyses of breast size and morphological variation.

**Conclusions:**

The modified hybrid plan, using an automatically calculated prescription dose for the right breast and taking into consideration the background dose from the left breast VMAT plan, showed comparable target coverage to that of the VMAT-only plan, and was superior for saving OARs. However, considering that VMAT can be adjusted according to the physician’s intention, further evaluation is needed for developing a better plan.

**Electronic supplementary material:**

The online version of this article (10.1186/s13014-019-1257-5) contains supplementary material, which is available to authorized users.

## Background

Breast cancer is the most common cancer in women, and 1–2% of these patients are diagnosed with synchronous bilateral breast cancer (SBBC) [1]. While the incidence of SBBC is low, it presents significantly poorer overall survival than that of unilateral breast cancer [2, 3]. There is no standard guideline for treating SBBC, and owing to an increasing demand for breast-conserving treatment in many cases, synchronous bilateral breast irradiation is commonly required.

Radiotherapy (RT) for unilateral breast cancer has used a tangential field with two-dimensional (2D) or three-dimensional conformal radiotherapy (3D-CRT). However, when the traditional field is applied to SBBC, overlapping of the RT fields could be inevitable, thereby compromising the target coverage. There is also a problem with the daily set-up of the patients, especially in the case of RT including regional node irradiation (RNI). Multiple isocenters are used in this method, so setting up the patient’s posture may often be inaccurate. Moreover, a larger treatment volume is required for SBBC, so the radiation dose to organs at risk (OARs), like the lung and heart, is considerable.

For treating such a complex target volume, recent trends have shown that intensity-modulated radiation therapy (IMRT) using helical tomotherapy or volumetric-modulated arc therapy (VMAT) are applied. When IMRT is used for SBBC, problems associated with the isocenter and junction can be solved. Because the irradiation beam in IMRT emits in many directions, a large volume of the lung and heart may be irradiated unnecessarily during bilateral breast treatment. Therefore, physicians have tried to reduce the dose to OARs by using hybrid-VMAT, limiting the beam direction, or using a static angle only in tomotherapy [4–6].

However, there is still no standard for the best plan, and in most previous studies, only RT for bilateral breasts without RNI was considered. In addition, the optimal RT plan considering the morphologic variation of patients (e.g. large/small breasts or a funnel-like chest) has not been reported. In this study, we evaluated the optimal RT plan for SBBC, especially treatment plans including RNI, and analyzed the strategies considering the patients’ morphologic variations.

## Methods

### Patients selection and planning objectives

In order to consider various breast sizes and types of breast surgery, we included 10 patients who received bilateral partial mastectomy (5 with small breasts and 5 with large breasts) and 5 patients who received a right breast partial mastectomy and a left breast total mastectomy. A small breast was defined as one where the depth from the nipple to the chest wall was < 3 cm in an axial computed tomography (CT) image, whereas a large breast was defined as one where the depth was > 3 cm (Additional file 1: Figure S1). The patients were immobilized in a supine position on a breast board with both arms raised, and planning CT was conducted.

The clinical target volume (CTV) for the breast, chest wall, and lymph node (LN) were contoured with reference to the Radiation Therapy Oncology Group contouring atlas [7]. To closely examine the irradiation dose to the heart, treatment of LN area was set to the left side. Thus, the CTV was defined as the volume that enveloped the bilateral whole breasts (or the right breast and left chest wall) and the left regional LN area, including the internal mammary, axillary level I, II, III, and supraclavicular LNs. The planning target volumes (PTVs) were obtained by 5 mm expansion in all directions from the CTVs and were also restricted to have a skin gap (trim) of 3 mm from the surface in the case of breast CTV. However, for patients who received a total mastectomy, we included the skin in the CTV without a gap. The whole lung, heart, and left anterior descending artery (LAD) were considered OARs.

The prescribed dose was 50.4 Gy at 1.8 Gy per fraction to the PTVs. Radiation boost to the tumor bed was not included in this study. The primary aim in RT planning was to deliver 95% of the prescribed dose in 95% of the PTV. For planning approval, PTV D_95%_ ≥ 47.8 Gy, a near-maximum dose (D_2%_) ≤ 55.4 Gy to the PTV, and a minimum dose to 95% of both PTVs that is not lower than 95% of their respective prescribed doses were required. As no distinct treatment protocol has been suggested for bilateral breast cancer, the OAR dose constraints guideline was established based on a study of several patients with unilateral breast cancer [8] and previous SBBC research [9]. The irradiation dose to the OARs was restricted as follows: the planning objectives were a mean lung dose of 15 Gy with V_20Gy_ < 30% (no more than 30% of the OAR volume receiving 20 Gy) for the lungs and V_25Gy_ < 20% for the heart. For the LAD, an objective of 25 Gy for the maximum dose (D_max_) was retained. A bolus was not used for any chest wall planning.

### Planning techniques

The RT plans were generated using RayStation software version 5.2 (RaySearch Laboratories, Sweden) and all plans were made for treatment on a Versa HD radiotherapy system (Elekta). The energy of 6 MV photon was used in all VMAT and 3D CRT plans.

### Using only VMAT for the whole PTV *(VMAT-only plan)*

For the VMAT-only plan, a single isocenter was used for the whole PTV. The plans used two pairs of partial arcs with a total length of 240° per arc (clockwise and counterclockwise), which consisted of a rotating beam on each breast. The starting gantry angle was 240° and the stop gantry angle was 120° for all patients. A fixed collimator angle is generally recommended to avoid an excessive inter-leaf leakage dose to the patient during VMAT. The impact is different depending on certain factors such as MLC type. With reference to previous papers on VMAT plans for the breast, the collimator and couch angles in this study were set at zero, and only a coplanar beam was used [4, 10, 11]. No monitor unit constraint was used in order to allow maximum OAR sparing.

### VMAT planning for the left breast followed by conventional 3D-tangential technique for the right breast *(primary hybrid plan)*

To cover the left PTV, one partial arc was used with a total length of 195°, a starting gantry angle between 300° and 305°, and a stop angle between 140° and 145°. After completing the VMAT plan for the left PTV, the right breast PTV was planned with a conventional tangential field, without considering the background dose of the previous VMAT plan. A total dose of 50.4 Gy with 28 fractions was prescribed at the prescription point of the right breast PTV.

### VMAT planning for the left breast followed by conventional tangential technique for the right breast, considering the background dose of previous VMAT planning *(modified hybrid plan)*

The VMAT plans for the left PTV in this method were the same as those of the primary hybrid plan. However, owing to the out-of-field dose distribution of the left VMAT plan in the right breast (Additional file 2: Figure S2), following the 3D plan may result in a high dose area if we use the prescribed dose of 50.4 Gy as is. Thus, we set the dose distribution of the previous left VMAT plan as a background dose when setting the new beam for the right breast. The multiple beam set planning in RayStation enables the optimization of the summed dose distribution for both VMAT and the 3D plan simultaneously. Objective functions can be assigned to each beam set dose, or to their sum. In this plan, the RayStation system calculated the background dose distributions in the right PTV, and automatically adjusted the dose of the prescription point to a final dose of 50.4 Gy.

### Analysis of plans

The plans were evaluated by dose-volume histogram (DVH) analysis. For PTV, the mean doses and values of V_105%_ and V_110%_ (the percentage of the PTV receiving at least 105 and 110% of the prescribed dose, respectively), D_max_, and D_mean_ (mean dose) were reported. The D_98%_ and D_2%_ (minimum dose to 98 and 2% of the PTV) were also reported. The conformity index (CI) was measured by BV_95%_ (defined below), and the dose homogeneity index (HI) was measured by D_5%_/D_95%_ [12]. CI and HI were calculated using the definitions below, and the closer the CI and HI values are to 1, the better the conformal coverage:

$$ \mathrm{CI}={\mathrm{BV}}_{95\%}/\mathrm{PTV}\ \mathrm{volume} $$

(BV_95%_ = body volume of the isodose of 95% of the prescribed dose)

$$ \mathrm{HI}={\mathrm{D}}_{5\%}/{\mathrm{D}}_{95\%} $$

(D_5%_ = minimum dose to 5% of the PTV, D_95%_ = minimum dose to 95_%_ of the PTV)

To evaluate the irradiated dose to OARs, the analysis included the mean dose and V_XGy_ (OAR volume receiving X Gy), depending upon the organ. For the lung, incidence of radiation pneumonitis (RP) was < 20% when the mean lung dose was less than approximately 20 Gy. With regard to V_dose_ threshold models, the risk of RP was < 20% for V20 < 30–35% or V5 < 60% with conventional fractionation [13–15]. The V_30Gy_, V_25Gy_, and D_mean_ for the heart were compared; V_20Gy_, V_10Gy_, D_max_, and D_mean_ for LAD were also compared. The Tukey test and one-way ANOVA were used to compare the PTV and OAR values of the three techniques. Statistical analyses were performed using SPSS software version 23 (IBM Corp, Armonk, NY). Differences were reported to be statistically significant at *p* < 0.05.

## Results

### Target dose distribution

The median pathologic tumor size of the 15 patients was 1.7 cm (range, 0.4–3.8 cm) for the left breast and 0.8 cm (range, 0.1–2.8 cm) for the right breast. Mean volume of the whole PTV was 988.6 ± 327.6 ml; the range of PTV volume was from 410.0 ml to 1765.5 ml. In the modified hybrid plan, the mean prescribed dose to the right PTV was 48.09 Gy (± 0.93 Gy) at the prescription point. The characteristics of the 15 patients in this study are presented in Additional file 3: Table S1.

**Fig. 1:**
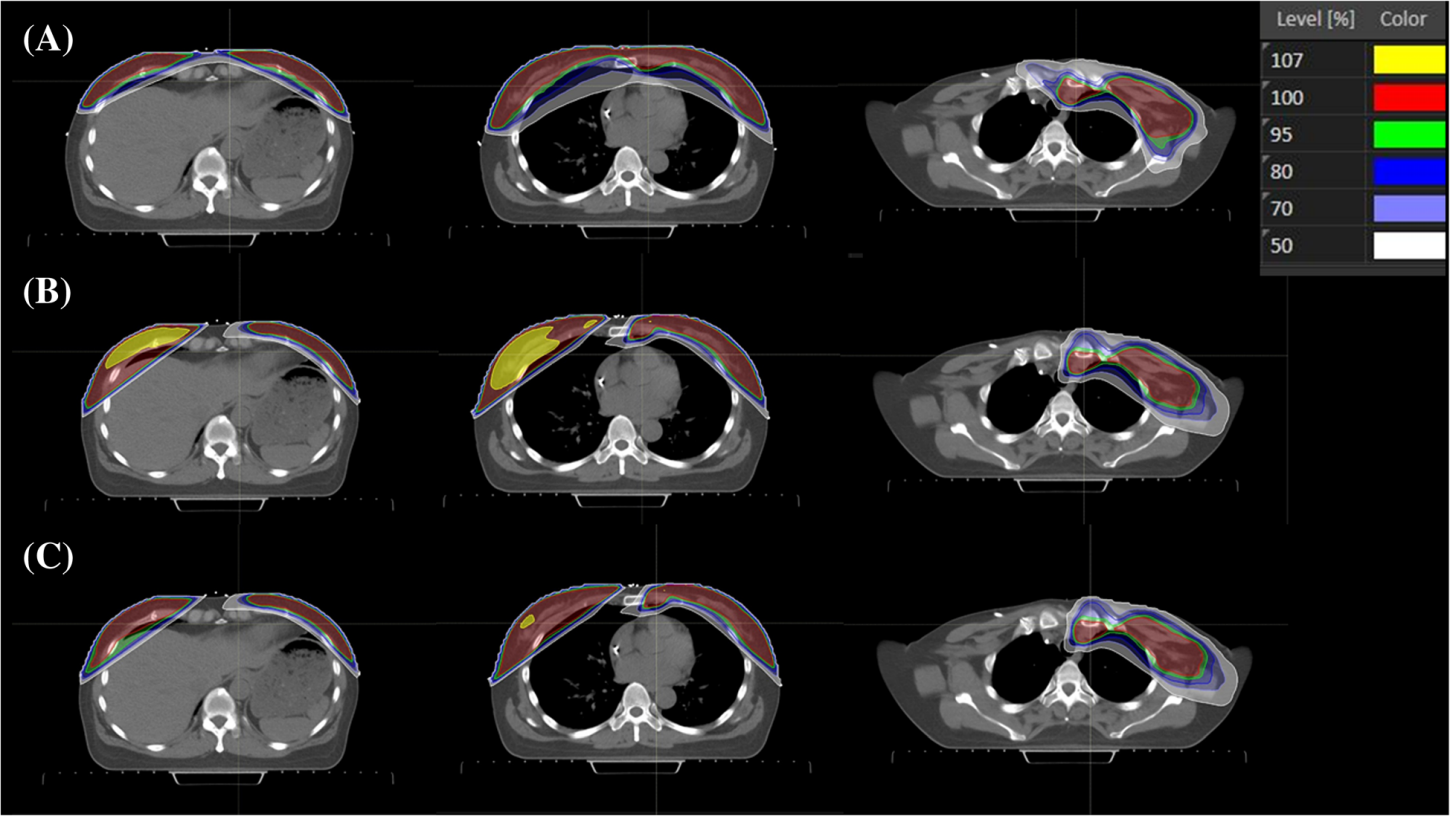
Isodose distribution of individual patients treated with various radiotherapy plans. The VMAT-only (**a**), primary hybrid (**b**), and modified hybrid plans (**c**) are shown. High dose areas (107% of the prescribed dose) are represented with yellow color

Table 1 shows the D_n%_ (dose (Gy) to n% of the PTV), V_m%_ (percentage of the PTV receiving ≥m% of prescribed dose), D_mean_, and D_max_. The primary hybrid plan showed the largest distribution of high-dose volume with a V_105%_ of 25.69% and V_110%_ of 6.37% in the PTV (*p* < 0.001). This plan also showed the highest D_max_ (59.21 ± 1.29 Gy), followed by the modified hybrid (56.2 ± 1.31 Gy) and VMAT-only plans (54 ± 1.14 Gy). The modified hybrid plan showed higher V_105%_ than the VMAT-only plan, but V_110%_ did not differ between the two plans. Figure 1 shows the isodose distribution of one patient treated with various radiotherapy plans.

**Table 1:** The comparison of planning target volume (PTV) coverage for VMAT only, primary hybrid, and modified hybrid plan using dosimetric parameters

	Treatment plans									
	VMAT only^1)^		Primary hybrid plan^2)^		Modified hybrid plan^3)^		*p* value	*p* ^a^		
PTV parameters	Mean	(SD)	Mean	(SD)	Mean	(SD)		1) vs 2)	1) vs 3)	2) vs 3)
V_95%_ (%)	96.7	(1.16)	97.1	(1.58)	95.3	(2.07)	0.026	0.821	0.079	0.02
V_105%_ (%)	0.3	(0.62)	26.7	(6.81)	6.4	(2.32)	< 0.001	< 0.001	0.001	< 0.001
V_110%_ (%)	0.0	(0)	6.2	(3.18)	0.4	(0.73)	< 0.001	< 0.001	0.87	< 0.001
D_98%_ (Gy)	44.6	(7.91)	47.5	(0.6)	47.7	(0.8)	0.145	0.225	0.181	0.992
D_2%_ (Gy)	54.0	(2.2)	54.4	(2.3)	54.0	(1.8)	0.81	0.824	0.998	0.859
D_mean_	50.3	(0.06)	51.6	(0.36)	50.5	(0.27)	< 0.001	< 0.001	0.106	< 0.001
D_max_	54.0	(1.14)	59.2	(1.29)	56.2	(1.31)	< 0.001	< 0.001	< 0.001	< 0.001
CI	1.50	(0.18)	1.46	(0.15)	1.49	(0.18)	0.862	0.856	0.986	0.927
HI	1.07	(0.02)	1.18	(0.03)	1.11	(0.03)	< 0.001	< 0.001	< 0.001	< 0.001

*p*^a^: Post-Hoc Analysis Using Tukey’s HSD test

The VMAT-only plan and modified hybrid plan showed comparable target coverage according to D_mean_ with mean values of 50.33 Gy and 50.53 Gy, respectively (*p* = 0.106), whereas the primary hybrid plan showed higher D_mean_. Although D_98%_ and D_2%_ were not significantly different between the 3 RT plans, D_98%_ for the VMAT-only plan was the lowest, with a large standard deviation, indicating that the D_98%_ of the VMAT-only plan is distributed widely around the mean value. There was also no significant difference in CI. However, the VMAT-only plan showed the best HI value (1.07 ± 0.02), followed by the modified hybrid (1.11 ± 0.03) and primary hybrid plans (1.18 ± 0.03) (*p* < 0.001).

Among the 10 patients who received bilateral partial mastectomy, 5 had small breasts and the other 5 had large breasts. In the subgroup analysis for patients with small breasts, the VMAT-only and modified hybrid plans showed equivalent target coverage in terms of D_mean_ (50.3 Gy vs 50.6 Gy, *p* = 0.057), whereas the primary hybrid plan showed a high D_mean_ value (51.6 Gy, p < 0.001) (Additional file 3: Table S2). HI (1.1 ± 0.01 p < 0.001) was best in the VMAT-only plan, while D_98%_, D_2%_, and CI showed no significant differences between the three plans.

For patients with large breasts, the VMAT-only and modified hybrid plans also showed comparable target volume coverage regarding D_mean_. Moreover, HI was not significantly different between the VMAT-only plan and modified hybrid plan. Additional analysis for those who received a left breast total mastectomy showed results consistent with those of the patients with large breasts. All three subgroup analyses by breast shape (small, large, and total mastectomy) revealed that D_max_, V_105%_, and V_110%_ were significantly higher in the primary hybrid plan than the other two plans (Additional file 3: Table S3).

### OAR dose distribution

Regarding the whole lung, the average D_mean_ of all cases was 12.6 ± 1.6 Gy, while V_5Gy_, V_10Gy_, and V_20Gy_ were 60.5 ± 9.1%, 36.1 ± 7%, and 21.9 ± 4.6%, respectively. Table 2 shows the statistical comparison of the OAR dose distribution for each treatment technique. All dosimetric parameters for the whole lung were significantly higher in the VMAT-only plans than in the two hybrid plans, while there was no significant difference between the hybrid plans.

**Table 2:** The comparison of the organs at risk (OAR) dose using dosimetric parameters

		Treatment plans									
		VMAT only^1)^		Primary hybrid plan^2)^		Modified hybrid plan^3)^			*p* ^a^		
		Mean	(SD)	Mean	(SD)	Mean	(SD)	*p* value	1) vs 2)	1) vs 3)	2) vs 3)
Whole Lung											
D_mean_	(Gy)	14.4	(0.67)	11.8	(1.19)	11.6	(1.18)	< 0.001	< 0.001	< 0.001	0.935
V_5Gy_	(%)	67.9	(9.63)	57.0	(6.34)	56.7	(6.38)	< 0.001	0.001	0.001	0.993
V_10Gy_	(%)	41.1	(7.9)	33.2	(4.39)	34.0	(5.6)	< 0.001	0.003	0.008	0.926
V_20Gy_	(%)	27.5	(2.02)	19.2	(2.42)	19.0	(2.4)	< 0.001	< 0.001	< 0.001	0.971
Heart											
D_mean_	(Gy)	13.2	(2.33)	8.0	(2.61)	8.0	(2.6)	< 0.001	< 0.001	< 0.001	1
V_25Gy_	(%)	11.5	(6.41)	4.0	(4.71)	4.0	(4.71)	0.001	0.001	0.001	1
V_30Gy_	(%)	6.4	(4.86)	2.4	(3.49)	2.4	(3.49)	0.005	0.024	0.023	1
LAD											
D_mean_	(Gy)	21.8	(3.73)	14.7	(5.25)	14.7	(5.27)	< 0.001	0.001	0.001	0.999
D_max_	(Gy)	33.7	(2.65)	33.0	(7.19)	32.9	(7.15)	0.566	0.947	0.933	0.999
V_10Gy_	(%)	92.7	(12.88)	56.7	(23.19)	54.9	(23.61)	< 0.001	< 0.001	< 0.001	0.97
V_20Gy_	(%)	59.8	(21.3)	29.2	(22.38)	29.0	(22.46)	0.001	0.001	0.001	1

**Fig. 2:**
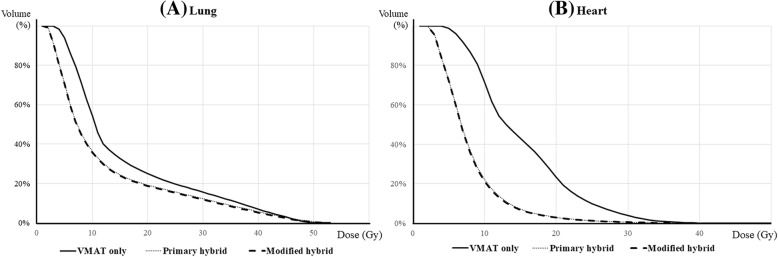
Comparison of dose volume for the planning target volume of the three radiotherapy plans. Histograms are shown for the (**a**) whole lung and (**b**) heart. The solid line represents the VMAT-only plan, the dotted line represents the primary hybrid plan, and the dashed line represents the modified hybrid plan (the two hybrid plans show almost the same dose distribution to the whole lung and heart)

The average heart dose in all cases was 9.7 ± 3.5 Gy. The irradiation dose to the heart was also highest in the VMAT-only plan, resulting in D_mean_ of 13.2 ± 2.3 Gy and V_25Gy_ of 11.5 ± 6.4%. Like in the whole lung, the primary and modified hybrid plans showed almost the same results for the heart dose. Compared to the VMAT-only plan, the hybrid plans had an approximately 5 Gy lower mean heart dose. No cases reported the heart volume receiving ≥40 Gy. Figure 2 shows the typical dose volume histogram for the whole lung (A) and heart (B). In both OARs, the VMAT-only plan showed a relatively high-volume distribution in both low- and high-dose regions.

For LAD, both the primary hybrid and modified hybrid plans showed lower D_mean_ than VMAT-only plans (14.7 Gy vs 21.8 Gy, *p* < 0.001). V_10Gy_ and V_20Gy_ were also significantly larger in VMAT-only plans. D_max_ showed no significance between the three RT plans.

In every subgroup analysis according to the surgery and breast shape, the VMAT-only plan showed significantly higher dose distribution in terms of mean lung dose, V_20Gy_ of lung, mean heart dose, and mean LAD dose (Additional file 3: Table S3).

Three patients in this study had funnel-like chests with convex lungs whose central lung distances (CLDs, distance from the chest wall to the edge of the field at the central axis) were larger than 2.5 cm, resulting in larger lung volume in the RT field in the conventional tangential plan (Additional file 4: Figure S3). We evaluated the lung dose distribution in these patients, and found that the V_5Gy_, V_10Gy_ and V_20Gy_ in the VMAT-only plan were greater than those in the two hybrid plans, as was D_mean_ (15.2 Gy, 11.9 Gy, and 12.0 Gy, respectively).

### Treatment efficiency

The VMAT-only plans resulted in a longer beam on time than those of the hybrid plans (115.3 s, 73.5 s, and 77.5 s, respectively, *p* < 0.001) (Table 3). The VMAT-only plans required 50% more time to deliver the radiation beam than the modified hybrid plan (115.3 ± 11.6 s vs. 7.5 ± 8.8 s, *p* < 0.001). All plans had similar MU values with no statistically significant differences.

**Table 3:** The comparison of the delivery parameters for VMAT only, primary hybrid, and modified hybrid plan

	VMAT only	Primary hybrid plan	Modified hybrid plan	p value
	Mean (SD)	Mean (SD)	Mean (SD)	
Beam on time (s)	115.3 (11.6)	73.5 (8.8)	77.5 (8.8)	< 0.001
MU segment	795.1 (131)	827.5 (59.2)	811.7 (67.3)	0.424

*Abbreviations*: *MU* monitor unit

## Discussion

In this study, the optimal RT plan for SBBC, especially when including regional LN, was investigated. The PTV is very large for treating the bilateral breasts and regional LN area. Thus, radiation exposure to OARs, like the lungs, increases compared to the PTV of unilateral breast cancer. A modified prescribed dose in a 3D plan for the right breast, considering the low dose distribution of the previous VMAT plan for the left breast and regional LN (i.e., the modified hybrid plan), was the best way to reduce the OAR dose while delivering the appropriate target volume coverage.

VMAT for the left breast and regional LN area improves target volume coverage and reduces treatment time and radiation dose to the lung, heart, and even LAD compared to the 3D plan [11, 16, 17]. However, despite these dosimetric advantages of VMAT, treatment for SBBC is more complicated. When using the arc in multiple directions to treat both breasts, a larger volume of the bilateral lungs is inevitably exposed to irradiation. To solve this problem, Subramanian et al. proposed the hybrid–VMAT (h-VMAT) technique [5]. The h-VMAT planning involves two steps. First, a field-in-field (FIF) forward planning setup with 80% of the prescription dose was planned for both breasts. The heart and lungs were spared using a high definition multi-leaf collimator. Second, the remaining 20% prescription dose for both the breasts was optimized using VMAT with three continuous arcs (arc length: 150°–210°) by keeping the dose delivered in a FIF arrangement as the base dose plan. With this method, the radiation dose for the lung was significantly decreased compared to that in the conventional VMAT plan. However, this strategy could be applied only for SBBC, but not including regional LN irradiation, and additional evaluation is needed.

The need to control and minimize the impact of breathing motion during IMRT for breast cancer has been investigated by several groups, and the breath-hold technique or breathing gating technique was identified as the most convenient and safe approach. As multileaf collimators without fully-opening jaws are used for VMAT, several set-up or positional errors could occur. Efforts are needed to reduce this gap, and Nicolini et al. suggested optimal VMAT planning for breast cancer [18]. Alternative images were generated with an artificial expansion of 10 mm from the body in the breast region and additional PTV was contoured to this image. The two treatment plans, which are performed on original and alternative images respectively, were optimized using original and alternative images. The proposed planning strategy could represent a robust approach that could account for moderate changes in target or body volume during the course of breast radiotherapy and also account for residual intrafractional respiratory motion in VMAT.

Here, we conducted a comparative study for the optimal RT plan for SBBC including regional LN irradiation. Among the previous studies on SBBC, Seppälä et al. and Boman et al. reported RT for SBBC including RNI [19, 20]. However, they only included axillary LNs and/or internal mammary LNs, whereas we included supraclavicular LNs; this resulted in a significant increase in lung dose. We implemented a hybrid plan using both VMAT and 3D-CRT instead of VMAT for this large target volume. Specifically, we used a 3D-tangential plan considering the widespread but low-dose area generated by previous VMAT plans on the left side. The dose coverage for the PTV was comparable to that of the VMAT-only plan, and the dose to OAR was significantly reduced. Especially for the lung, VMAT plans generally produced a larger volume in which a low dose of radiation is distributed, compared to that of a tangential field [11]. Considering the mean lung dose (MLD) of 6–16 Gy in previous SBBC studies [4, 9, 19, 21], our results were comparable to previous reports, despite the inclusion of the regional LN area such as the internal mammary chain and supraclavicular area.

In breast cancer, evidence is accumulating that RT can increase the risk of heart disease [22], and an increased risk of stenosis in the LAD for left-sided RT compared to right-sided RT has been reported [23]. Contemporary techniques usually deliver lower mean doses to the heart than they did in the past, but some parts of the heart may still receive high doses including the LAD, which is located near the left breast and may receive a high dose in RT for left breast cancer. Although Darby et al. suggested that increasing the mean heart dose could increase the incidence of ischemic heart disease [22, 24], specific thresholds have been defined for neither the dose in the heart nor the dose in the LAD, according to several authors [25]. Since no threshold doses for the heart and LAD are available and the clinical effect of low doses is not completely clear, we think the best clinical practice would be to keep the dose in the heart and LAD as low as achievable. In this study, the mean dose and V_xGy_ of the heart and LAD were also highest in the bilateral VMAT plan. Hybrid techniques using VMAT +3D-CRT can reduce the heart dose, and additional methods such as deep inspiration breath holding can reduce the radiation exposure to cardiac structures.

Although the hybrid plans showed shorter beam on time than that of the VMAT-only plan, hybrid plans would consume more treatment time due to the movement of the isocenter. Moreover, the setup during treatment could result in some errors involving factors such as quality of treatment and patients’ satisfaction. However, previous studies reported the feasibility of the two-isocenter technique in treating SBBC [20]. The two-isocenter technique induced 2–5 mm of errors but no clinically significant change of dose coverage. The studies also emphasized that the two-isocenter VMAT technique could reduce the mean dose for the lung and heart better than the single isocenter technique.

There are several limitations in this study. First, we only considered the regional LN in the left side. For bilateral regional LN, the VMAT-plan may be the best, as the AP field for supraclavicular LN in 3D CRT generates a large amount of upper lung dose. To counter this issue further evaluation is needed based on the two-isocenter VMAT suggested by Boman et al. [18]. Second, this study was conducted using an Asian population, and the criterion for classifying large breasts was extremely small when compared to those for Caucasian populations; therefore, further studies based on Caucasian populations are needed. In addition, only the beam delivery time was calculated, and it was the longest when the VMAT-only plan was performed. In actual patient treatment, however, two isocenters are used in the hybrid plan, which may lead to a longer patient set-up time. Therefore, further evaluation is needed to confirm whether hybrid plans are more efficient than the VMAT-only plan in clinical use. Moreover, since VMAT plans can be adjusted according to the physician’s intention, better plans could be generated by modifying the geometry of the beams.

## Conclusions

The modified hybrid plan using VMAT + modified 3D-CRT is best when considering both PTV coverage and protection of OARs. To identify the clinical efficacy of the modified hybrid plan in terms of oncologic outcomes and treatment toxicities, advanced long-term follow-up studies with a large number of patients are needed. In addition, there is no standard guideline for the RT of SBBC including RNI, so it is also necessary to determine whether there are additional RT strategies beyond the method presented in this study.

To reach a compromise between dosimetric and therapeutic efficiency, and to improve the treatment of patients with SBBC, further studies of the optimal RT planning method, with a larger number of participants, should be performed.

## Additional files

Additional file 1: **Figure S1.** A large breast was defined as the one where the deputh was > 3 cm in an axial computed tomography (CT) image (A), whereas a small breast was defined as one where the depth from the nipple to the chest wall was < 3 cm (B). (C) is the CT image of patient who received left total mastectomy. (TIF 341 kb)

Additional file 2: **Figure S2.** Isodose distribution of VMAT plan of left PTV. Out-field dose distribution of the left VMAT plan is observed in right breast. (TIF 495 kb)

Additional file 3: **Table S1.** Patients and tumor characteristics. **Table S2.** The comparison of planning target volume (PTV) coverage for VMAT only, primary hybrid, and modified hybrid plans according to the patients’ morphologic variations using dosimetric parameters. **Table S3.** The comparison of the organs at risk dose according to the patients’ morphologic variations using dosimetric parameters (DOCX 28 kb)

Additional file 4: **Figure S3.** A computed tomography image of a patients whose central lung distance (CLDs, distance from the chest wall to the edge of the field at the central axis) is larger than 2.5 cm. (TIF 318 kb)

## Abbreviations

2D: two-dimensional
3D: three-dimensional
BV95%: body volume of the isodose of 95% of the prescribed dose
CI: conformity index
CRT: conformal radiotherapy
CT: computed tomography
CTV: clinical target volume
D_max_: maximum dose
D_mean_: mean dose
HI: homogeneity index
IMRT: intensity-modulated radiation therapy
LAD: left anterior descending artery
LN: lymph node
OAR: organ at risk
PTV: planning target volume
RNI: regional node irradiation
RT: radiotherapy
SBBC: synchronous bilateral breast cancer
VMAT: volumetric-modulated arc therapy
V_X%_: percentage of the PTV receiving at least X% of the prescribed dose
V_XGy_: OAR volume receiving a dose of X Gy
