# Supplementary figures and images for: Evaluation of optimal treatment planning for radiotherapy of synchronous bilateral breast cancer including regional lymph node irradiation
Source: Radiat Oncol. 2019 Apr 1;14:56. doi: 10.1186/s13014-019-1257-5 (PMC6444509; doi:10.1186/s13014-019-1257-5)

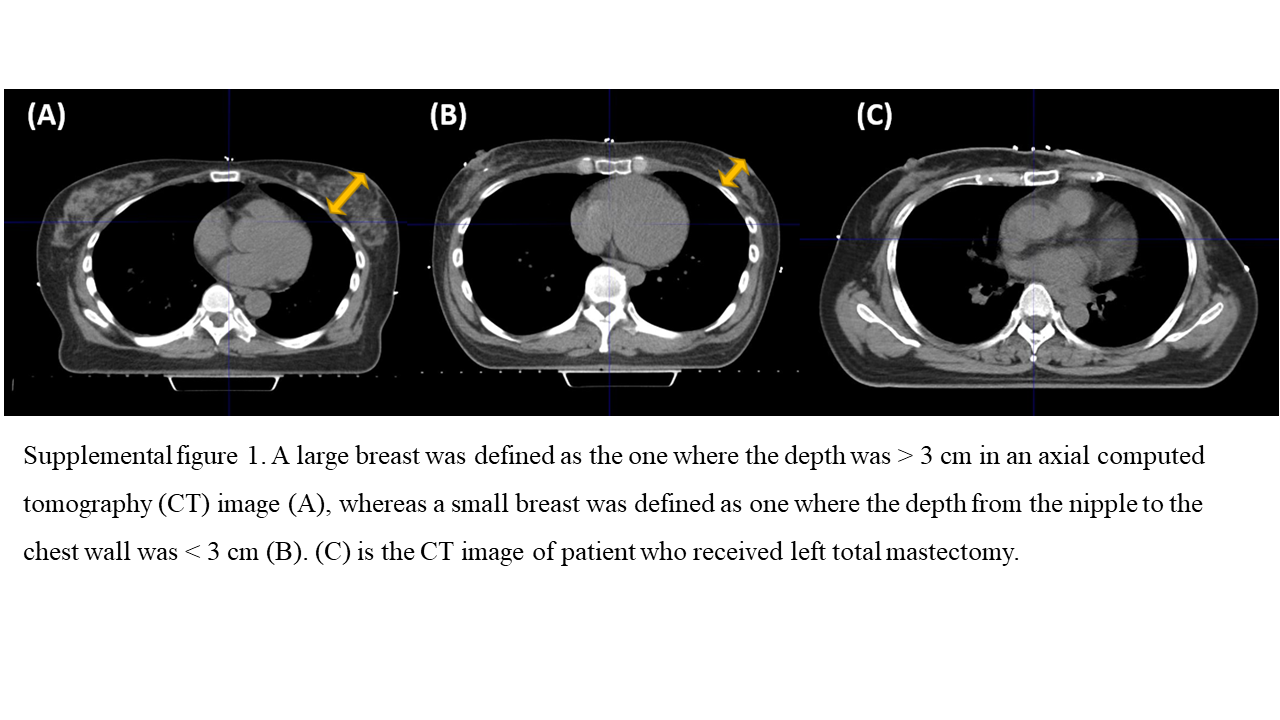

Supplement: Supplementary file 1 — Figure S1. A large breast was defined as the one where the deputh was > 3 cm in an axial computed tomography (CT) image (A), whereas a small breast was defined as one where the depth from the nipple to the chest wall was < 3 cm (B). (C) is the CT image of patient who received left total mastectomy. (TIF 341 kb) [file 13014_2019_1257_MOESM1_ESM.tif]

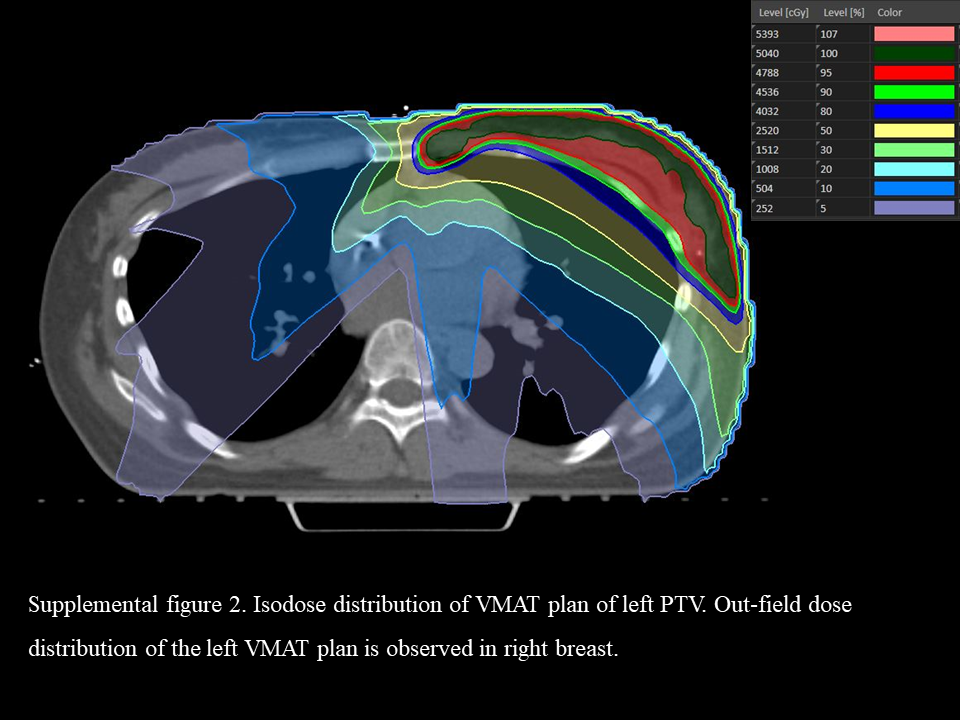

Supplement: Supplementary file 2 — Figure S2. Isodose distribution of VMAT plan of left PTV. Out-field dose distribution of the left VMAT plan is observed in right breast. (TIF 495 kb) [file 13014_2019_1257_MOESM2_ESM.tif]

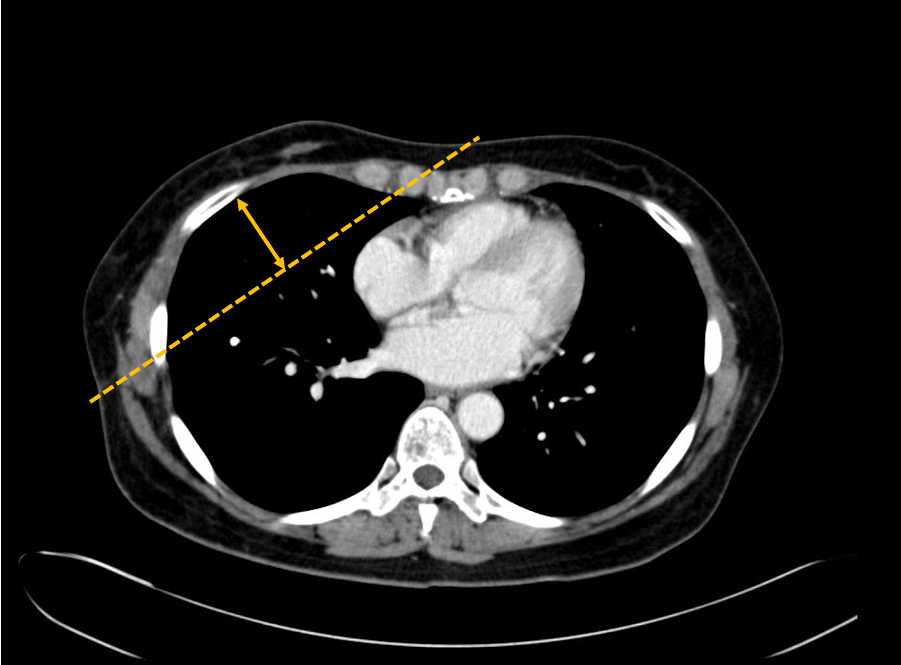

Supplement: Supplementary file 4 — Figure S3. A computed tomography image of a patients whose central lung distance (CLDs, distance from the chest wall to the edge of the field at the central axis) is larger than 2.5 cm. (TIF 318 kb) [file 13014_2019_1257_MOESM4_ESM.tif]
