# Supplementary material for: Evaluation of optimal treatment planning for radiotherapy of synchronous bilateral breast cancer including regional lymph node irradiation
Source: Radiat Oncol. 2019 Apr 1;14:56. doi: 10.1186/s13014-019-1257-5 (PMC6444509; doi:10.1186/s13014-019-1257-5)
Supplement: Supplementary file 3 — Table S1. Patients and tumor characteristics. Table S2. The comparison of planning target volume (PTV) coverage for VMAT only, primary hybrid, and modified hybrid plans according to the patients’ morphologic variations using dosimetric parameters. Table S3. The comparison of the organs at risk dose according to the patients’ morphologic variations using dosimetric parameters (DOCX 28 kb) [file 13014_2019_1257_MOESM3_ESM.docx]

Table S1. Patients and tumor characteristics

| Patients | Size | Age | BMI^1^ | Tumor size (cm) | | T stage | | N stage | | | Breast / chest wall CTV volume (ml) | | Whole PTV volume | Modified prescribed dose  for right breast  (% of prescribed dose) | CLD^2^ in tangential plan |
| --- | --- | --- | --- | --- | --- | --- | --- | --- | --- | --- | --- | --- | --- | --- | --- |
|  |  |  | (kg/m^2^) | Left | Right | Left | Right | | Left | Right | Left | Right | (ml) |  | (cm) |
| 1 | Small | 42 | 20.2 | 2.2 | 1.2 | 2 | 1c | | 0 | 0 | 276.4 | 260.1 | 670.4 | 97.1% | 1.8 |
| 2 | Small | 43 | 22.7 | 0.7 | 1.3 | 1b | Tis | | 1 | 0 | 338.1 | 365.6 | 852.4 | 95.9% | 1.5 |
| 3 | Small | 54 | 22.9 | 1.1 | 1 | 1c | 1c | | 0 | 0 | 232.3 | 271.8 | 711.2 | 95.7% | 2.5 |
| 4 | Small | 54 | 22.3 | 0.5 | 0.1 | Tis | Tis | | 0 | 0 | 361.7 | 396.1 | 868.3 | 96.2% | 1.6 |
| 5 | Small | 48 | 18 | 0.4 | 2.5 | Tis | Tis | | 0 | 0 | 146.5 | 157.4 | 410.0 | 98.9% | 1.5 |
| 6 | Large | 46 | 22.3 | 1.2 | 0.7 | 1c | 1b | | 0 | 1 | 342.5 | 442.2 | 880.4 | 96.2% | 0.5 |
| 7 | Large | 56 | 22.8 | 2.4 | 0.3 | Tis | Tis | | 0 | 0 | 504.5 | 618.7 | 1235.3 | 92.1% | 1.8 |
| 8 | Large | 42 | 28.3 | 0.9 | 0.9 | 1b | 1b | | 0 | 0 | 723.2 | 644.8 | 1503.4 | 96.7% | 1.8 |
| 9 | Large | 65 | 27.7 | 2.5 | 0.3 | Tis | Tis | | 0 | 1 | 532.9 | 528.9 | 1246.2 | 94.6% | 2.6 |
| 10 | Large | 44 | 23.2 | 1.3 | 2.8 | 1c | 2 | | 1 | 0 | 432.6 | 416.8 | 962.8 | 96.3% | 2.3 |
| 11 | TM^3^ | 47 | 22.5 | 2.2 | 0.6 | 2 | Tis | | 0 | 0 | 386.7 | 304.5 | 888.6 | 91.8% | 2.7 |
| 12 | TM | 66 | 25.2 | 2 | 0.3 | 2 | 1a | | 1 | 0 | 316.8 | 505.8 | 983.5 | 93.7% | 1.3 |
| 13 | TM | 55 | 28.9 | 1.8 | 0.8 | 1c | 1b | | 2 | 0 | 281.4 | 476.7 | 957.8 | 96.1% | 0.5 |
| 14 | TM | 45 | 27.7 | 1.7 | 0.3 | 1c | 1a | | 1 | 1 | 776.9 | 815.1 | 1765.5 | 95.6% | 1.2 |
| 15 | TM | 57 | 24.3 | 3.8 | 1.9 | 2 | 1c | | 0 | 0 | 374.4 | 393.3 | 892.6 | 94.5% | 2 |

*Abbreviations:* BMI^1^= body mass index, CLD^2^= central lung distance, TM^3^= total mastectomy

Table S2. The comparison of planning target volume (PTV) coverage for VMAT only, primary hybrid, and modified hybrid plans according to the patients’ morphologic variations using dosimetric parameters

|  |  | Treatment plans | | | | | | |  |  |  |  |
| --- | --- | --- | --- | --- | --- | --- | --- | --- | --- | --- | --- | --- |
|  |  | VMAT only^1)^ | | Primary hybrid plan^2)^ | | Modified hybrid plan^3)^ | | *p* value |  | p^a^ |  |  |
| Subgroup | Parameters | Mean | (SD) | Mean | (SD) | Mean | (SD) |  |  | 1) vs 2) | 1) vs 3) | 2) vs 3) |
| Small breast | V_95%_ (%) | 97.1 | (1.35) | 97.4 | (0.74) | 95.6 | (0.87) | 0.031 |  | 0.875 | 0.082 | 0.035 |
| (n=5) | V_105%_ (%) | 0.1 | (0.09) | 24.9 | (0.62) | 9.6 | (1.30) | <0.001 |  | <0.001 | 0.037 | <0.001 |
|  | V_110%_ (%) | 0.0 | (0.00) | 6.7 | (4.01) | 0.9 | (1.14) | 0.002 |  | 0.002 | 0.834 | 0.006 |
|  | D_98%_ (Gy) | 47.6 | (0.84) | 42.3 | (0.40) | 47.9 | (0.77) | 0.421 |  | 0.764 | 0.792 | 0.389 |
|  | D_2%_ (Gy) | 55.3 | (2.23) | 54.7 | (2.02) | 53.6 | (1.69) | 0.446 |  | 0.899 | 0.425 | 0.679 |
|  | D_mean_ | 50.3 | (0.00) | 51.6 | (0.31) | 50.6 | (0.13) | <0.001 |  | <0.001 | 0.057 | <0.001 |
|  | D_max_ | 53.3 | (1.42) | 59.1 | (1.14) | 56.3 | (1.42) | < 0.001 |  | <0.001 | 0.003 | 0.006 |
|  | CI | 1.6 | (0.18) | 1.5 | (0.18) | 1.6 | (0.25) | 0.797 |  | 0.791 | 0.884 | 0.982 |
|  | HI | 1.1 | (0.01) | 1.2 | (0.02) | 1.1 | (0.03) | < 0.001 |  | <0.001 | 0.005 | 0.004 |
|  |  |  |  |  |  |  |  |  |  |  |  |  |
| Large breast | V_95%_ (%) | 96.5 | (1.90) | 97.6 | (1.31) | 97.2 | (1.32) | 0.553 |  | 0.537 | 0.744 | 0.935 |
| (n=5) | V_105%_ (%) | 0.5 | (1.06) | 23 | (2.97) | 5.5 | (2.81) | <0.001 |  | <0.001 | 0.019 | <0.001 |
|  | V_110%_ (%) | 0.0 | (0.00) | 3.8 | (2.41) | 0.08 | (0.08) | 0.001 |  | 0.02 | 0.995 | 0.003 |
|  | D_98%_ (Gy) | 43.9 | (10.01) | 47.0 | (0.35) | 47.6 | (0.99) | 0.580 |  | 0.690 | 0.587 | 0.984 |
|  | D_2%_ (Gy) | 53.8 | (2.71_ | 54.8 | (2.11) | 54.9 | (2.06) | 0.344 |  | 0.772 | 0.745 | 0.999 |
|  | D_mean_ | 50.3 | (0.05) | 51.5 | (0.13) | 50.5 | (0.19) | <0.001 |  | <0.001 | 0.082 | <0.001 |
|  | D_max_ | 54.3 | (1.54) | 58.9 | (0.55) | 56 | (0.94) | <0.001 |  | <0.001 | 0.064 | 0.004 |
|  | CI | 1.5 | (0.18) | 1.4 | (0.05) | 1.5 | (0.11) | 0.335 |  | 0.377 | 0.994 | 0.428 |
|  | HI | 1.1 | (0.03) | 1.2 | (0.01) | 1.1 | (0.04) | <0.001 |  | >0.001 | 0.078 | 0.004 |
|  |  |  |  |  |  |  |  |  |  |  |  |  |
| Total mastectomy | V_95%_ (%) | 96.6 | (1.84) | 96.4 | (2.32) | 93.1 | (1.36) | 0.022 |  | 0.981 | 0.032 | 0.044 |
| (n=5) | V_105%_ (%) | 0.2 | (0.28) | 32.2 | (7.56) | 7.1 | (2.74) | <0.001 |  | <0.001 | 0.087 | <0.001 |
|  | V_110%_ (%) | 0.0 | (0.00) | 8.0 | (1.36) | 0.1 | (0.13) | <0.001 |  | <0.001 | 0.987 | <0.001 |
|  | D_98%_ (Gy) | 42.4 | (9.91) | 48.1 | (0.27) | 47.5 | (0.82) | 0.268 |  | 0.298 | 0.375 | 0.984 |
|  | D_2%_ (Gy) | 52.8 | (0.71) | 53.7 | (3.01) | 53.5 | (1.69) | 0.765 |  | 0.764 | 0.852 | 0.985 |
|  | D_mean_ | 50.34 | (0.09) | 51.7 | (0.58) | 50.4 | (0.42) | <0.001 |  | 0.001 | 0.95 | 0.001 |
|  | D_max_ | 54.1 | (0.97) | 59.6 | (1.97) | 50.1 | (1.74) | 0.001 |  | <0.001 | 0.156 | 0.013 |
|  | CI | 1.4 | (0.15) | 1.5 | (0.15) | 1.4 | (0.18) | 0.001 |  | 0.628 | 0.92 | 0.85 |
|  | HI | 1.1 | (0.02) | 1.2 | (0.04) | 1.1 | (0.06) | 0.652 |  | 0.001 | 0.178 | 0.015 |

Table S3. The comparison of the organs at risk dose according to the patients’ morphologic variations using dosimetric parameters

|  |  |  |  | Treatment plans | | | | | | |  |  |  |  |
| --- | --- | --- | --- | --- | --- | --- | --- | --- | --- | --- | --- | --- | --- | --- |
|  |  |  |  | VMAT only^1)^ | | Primary hybrid plan^2)^ | | Modified hybrid plan^3)^ | |  |  | *p*^a^ |  |  |
|  |  |  |  | Mean | (SD) | Mean | (SD) | Mean | (SD) | *p* value |  | 1) vs 2) | 1) vs 3) | 2) vs 3) |
| Small breast | Whole Lung | D_mean_ | (Gy) | 14.1 | (0.66) | 10.9 | (0.75) | 10.8 | (0.74) | <0.001 |  | <0.001 | <0.001 | 0.962 |
|  |  | V_5Gy_ | (%) | 64.8 | (8.48) | 52.2 | (5.95) | 51.9 | (5.91) | <0.001 |  | 0.001 | 0.001 | 0.997 |
|  |  | V_10Gy_ | (%) | 39.2 | (13.83) | 31.2 | (2.54) | 30.9 | (2.54) | <0.001 |  | 0.001 | 0.001 | 0.999 |
|  |  | V_20Gy_ | (%) | 25.5 | (1.15) | 18.2 | (1.25) | 18.1 | (1.25) | <0.001 |  | <0.001 | <0.001 | 0.97 |
|  | Heart | D_mean_ | (Gy) | 13.1 | (2.08) | 5.8 | (0.92) | 5.8 | (0.89) | <0.001 |  | <0.001 | <0.001 | 1 |
|  |  | V_25Gy_ | (%) | 8.2 | (4.41) | 0.7 | (0.78) | 0.7 | (0.76) | 0.001 |  | 0.002 | 0.002 | 1 |
|  |  | V_30Gy_ | (%) | 5.4 | (5.42) | 0.3 | (0.42) | 0.3 | (0.41) | 0.04 |  | 0.064 | 0.063 | 1 |
|  | LAD | D_mean_ | (Gy) | 21.7 | (3.63) | 10.7 | (4.76) | 10.6 | (4.69) | 0.002 |  | 0.005 | 0.005 | 0.999 |
|  |  | D_max_ | (Gy) | 34.3 | (1.64) | 29.2 | (9.12) | 28.9 | (8.84) | 0.458 |  | 0.54 | 0.505 | 0.998 |
|  |  | V_10Gy_ | (%) | 98.4 | (2.49) | 36.2 | (24.52) | 35.5 | (23.86) | <0.001 |  | 0.001 | 0.001 | 0.998 |
|  |  | V_20Gy_ | (%) | 59.9 | (24.9) | 14.0 | (20.72) | 13.6 | (20.64) | 0.009 |  | 0.017 | 0.016 | 0.999 |
|  |  |  |  |  |  |  |  |  |  |  |  |  |  |  |
| Large breast | Whole Lung | D_mean_ | (Gy) | 14.5 | (0.90) | 12.2 | (0.64) | 12.1 | (0.73) | <0.001 |  | 0.001 | 0.001 | 0.995 |
|  |  | V_5Gy_ | (%) | 65.6 | (12.26) | 58.9 | (3.20) | 58.4 | (3.35) | 0.284 |  | 0.372 | 0.327 | 0.995 |
|  |  | V_10Gy_ | (%) | 41.1 | (3.29) | 33.2 | (2.91) | 36.3 | (6.54) | 0.052 |  | 0.044 | 0.261 | 0.54 |
|  |  | V_20Gy_ | (%) | 27.9 | (1.74) | 20.1 | (1.77) | 19.9 | (1.78) | <0.001 |  | <0.001 | <0.001 | 0.986 |
|  | Heart | D_mean_ | (Gy) | 12.0 | (2.59) | 7.8 | (2.03) | 7.8 | (2.06) | 0.018 |  | 0.032 | 0.03 | 1 |
|  |  | V_25Gy_ | (%) | 8.3 | (5.40) | 2.8 | (2.61) | 2.8 | (2.61) | 0.062 |  | 0.094 | 0.094 | 1 |
|  |  | V_30Gy_ | (%) | 3.7 | (3.91) | 1.5 | (1.58) | 1.4 | (1.69) | 0.31 |  | 0.384 | 0.366 | 0.999 |
|  | LAD | D_mean_ | (Gy) | 19.8 | (2.31) | 14.9 | (3.34) | 14.9 | (3.34) | 0.039 |  | 0.062 | 0.062 | 1 |
|  |  | D_max_ | (Gy) | 31.3 | (2.26) | 33.1 | (7.41) | 33.1 | (7.41) | 0.875 |  | 0.896 | 0.896 | 1 |
|  |  | V_10Gy_ | (%) | 92.1 | (13.68) | 61.5 | (12.49) | 57.0 | (16.26) | 0.004 |  | 0.014 | 0.0066 | 0.875 |
|  |  | V_20Gy_ | (%) | 47.1 | (15.97) | 29.4 | (20.64) | 29.4 | (20.64) | 0.245 |  | 0.308 | 0.306 | 1 |
|  |  |  |  |  |  |  |  |  |  |  |  |  |  |  |
| Total | Whole Lung | D_mean_ | (Gy) | 14.7 | (0.42) | 12.2 | (1.63) | 12.0 | (1.58) | 0.015 |  | 0.031 | 0.023 | 0.985 |
| mastectomy |  | V_5Gy_ | (%) | 63.6 | (3.56) | 60.0 | (7.13) | 59.7 | (7.21) | 0.558 |  | 0.634 | 0.598 | 0.998 |
|  |  | V_10Gy_ | (%) | 43.0 | (2.21) | 35.2 | (6.51) | 34.9 | (6.44) | 0.061 |  | 0.101 | 0.086 | 0.995 |
|  |  | V_20Gy_ | (%) | 29.0 | (1.38) | 19.3 | (3.71) | 19.0 | (3.64) | <0.001 |  | 0.001 | 0.001 | 0.993 |
|  | Heart | D_mean_ | (Gy) | 14.5 | (2.02) | 10.5 | (2.18) | 10.5 | (2.18) | 0.018 |  | 0.032 | 0.032 | 1 |
|  |  | V_25Gy_ | (%) | 17.8 | (4.51) | 8.3 | (5.65) | 8.3 | (5.67) | 0.022 |  | 0.038 | 0.037 | 1 |
|  |  | V_30Gy_ | (%) | 10.1 | (3.24) | 5.5 | (4.65) | 5.5 | (4.61) | 0.172 |  | 0.229 | 0.226 | 1 |
|  | LAD | D_mean_ | (Gy) | 24.1 | (4.34) | 18.7 | (4.79) | 18.7 | (4.79) | 0.025 |  | 0.025 | 0.032 | 1 |
|  |  | D_max_ | (Gy) | 35.5 | (2.23) | 36.8 | (2.73) | 36.8 | (2.73) | 0.688 |  | 0.733 | 0.733 | 1 |
|  |  | V_10Gy_ | (%) | 87.6 | (17.71) | 72.3 | (16.32) | 72.2 | (16.39) | 0.286 |  | 0.353 | 0.347 | 1 |
|  |  | V_20Gy_ | (%) | 71.2 | (19.47) | 44.0 | (185.30) | 44.0 | (18.30) | 0.062 |  | 0.095 | 0.094 | 1 |

Abbreviations: LAD= left anterior descending artery
